# Supplementary material for: Clustering of NLRP3 induced by membrane or protein scaffolds promotes inflammasome assembly
Source: Nat Commun. 2025 May 27;16:4887. doi: 10.1038/s41467-025-60277-4 (PMC12117088; doi:10.1038/s41467-025-60277-4)
Supplement: Supplementary file 1 — Supplementary Information [file 41467_2025_60277_MOESM1_ESM.pdf]

Supplementary Information

Clustering of NLRP3 induced by membrane or protein scaffolds promotes inflammasome assembly

Elvira Boršić[1,2], Taja Železnik Ramuta[1], Sara Orehek[1], Mateja Erdani Kreft[3], Matthias Geyer[4], Roman Jerala[1,5] and Iva Hafner-Bratkovič[1,3,6]*

[1]Department of Synthetic Biology and Immunology, National Institute of Chemistry, Ljubljana, Slovenia.

[2]Interdisciplinary Doctoral Study of Biomedicine, Faculty of Medicine, University of Ljubljana, Ljubljana, Slovenia.

[3]Institute of Cell Biology, Faculty of Medicine, University of Ljubljana, Ljubljana, Slovenia.

[4]Institute of Structural Biology, University Clinics Bonn, University of Bonn, Bonn, Germany.

[5]Centre for the Technologies of Gene and Cell Therapy, National Institute of Chemistry, Ljubljana, Slovenia

[6]EN-FIST Centre of Excellence, Ljubljana, SI-1000, Slovenia.

*Corresponding author. Email: iva.hafner@ki.si

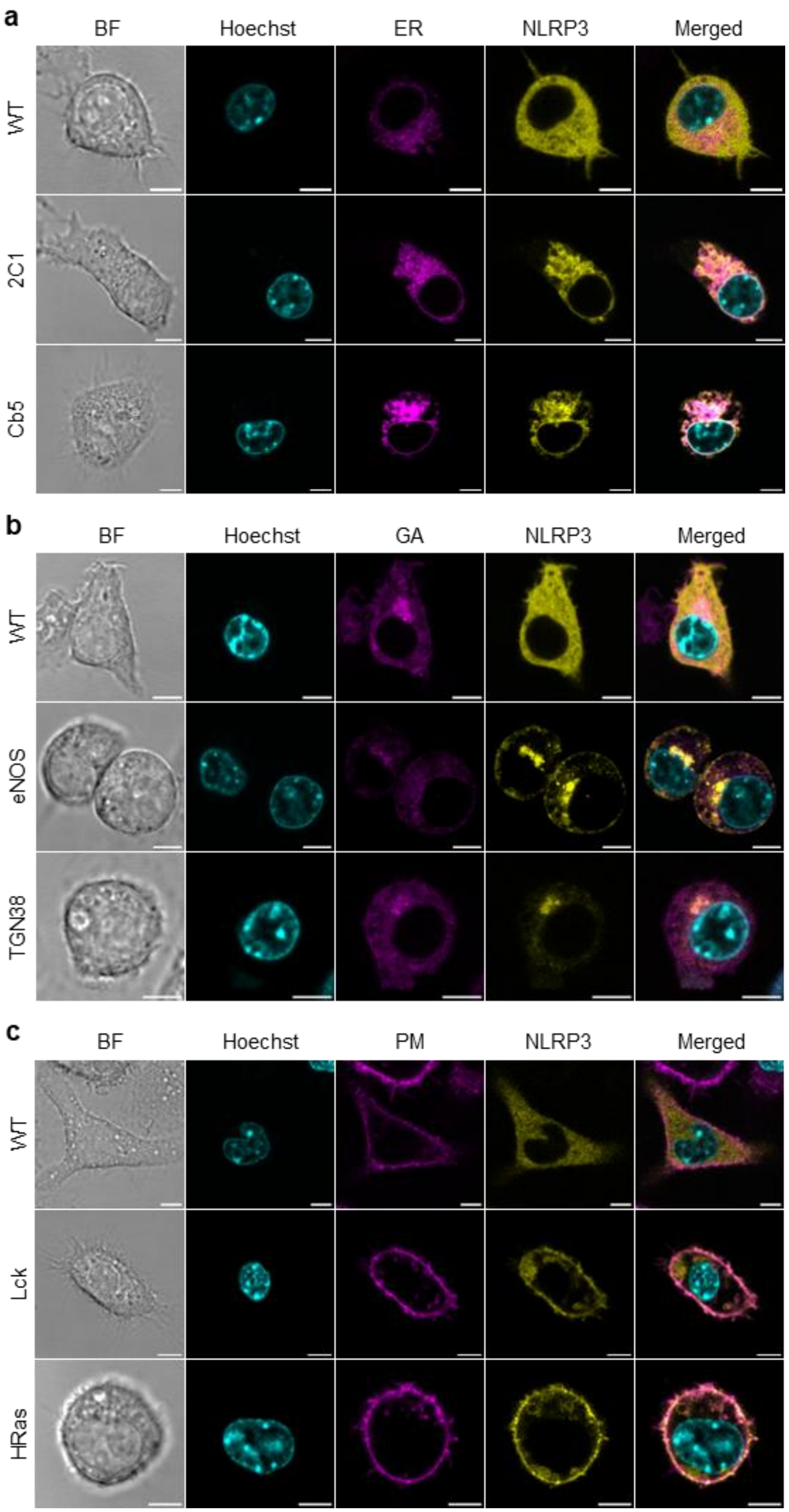

Supplementary Fig. 1. **Designed NLRP3 variants are enriched at ER, GA, and plasma membrane. a-c** NLRP3-KO iBMDMs stably expressing indicated wild-type (WT) or organelle-enriched NLRP3-YFP variants were primed with 100 ng/ml LPS and treated with 1 μg/ml doxycycline (Dox) for 12 hours, followed by 30-minute staining with ER-Tracker Red for endoplasmic reticulum (ER) (**a**), BODIPY TR C_5 for Golgi apparatus (GA) (**b**) or Cholera toxin subunit B, Alexa Fluor

647 for plasma membrane (PM) (**c**). Nuclei were stained with Hoechst 33342. Scale bar, 5 µm. BF, brightfield. Images are representative of three independent experiments.

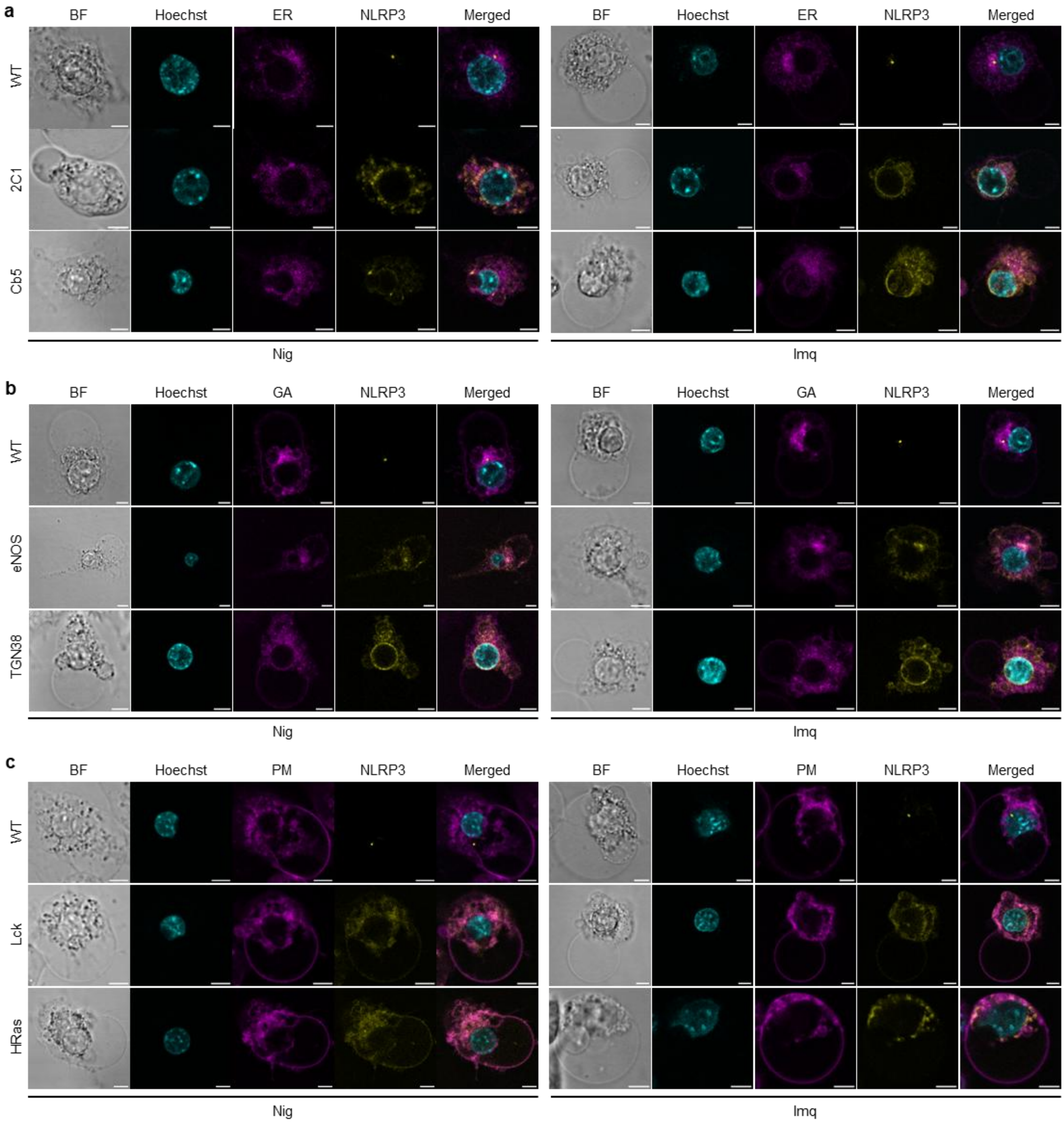

Supplementary Fig. 2. **Subcellular location of NLRP3 variants is unaffected by canonical triggers**. **a-c** NLRP3-KO iBMDMs expressing indicated NLRP3-YFP variants were primed with 100 ng/ml LPS and 1 µg/ml doxycycline (Dox) for 12 hours, after which cells were stained for 30 minutes with ER-Tracker Red for endoplasmic reticulum (ER) (**a**), BODIPY TR C_5 for Golgi apparatus (GA) (**b**) or Cholera toxin subunit B, Alexa Fluor 647 for plasma membrane (PM) (**c**). Cells were treated with nigericin

or imiquimod and imaged within 1 or 1.5 hours post-treatment, respectively. Nuclei were stained with Hoechst 33342. Scale bar, 5 μm. BF, brightfield. Images are representative of three independent experiments.

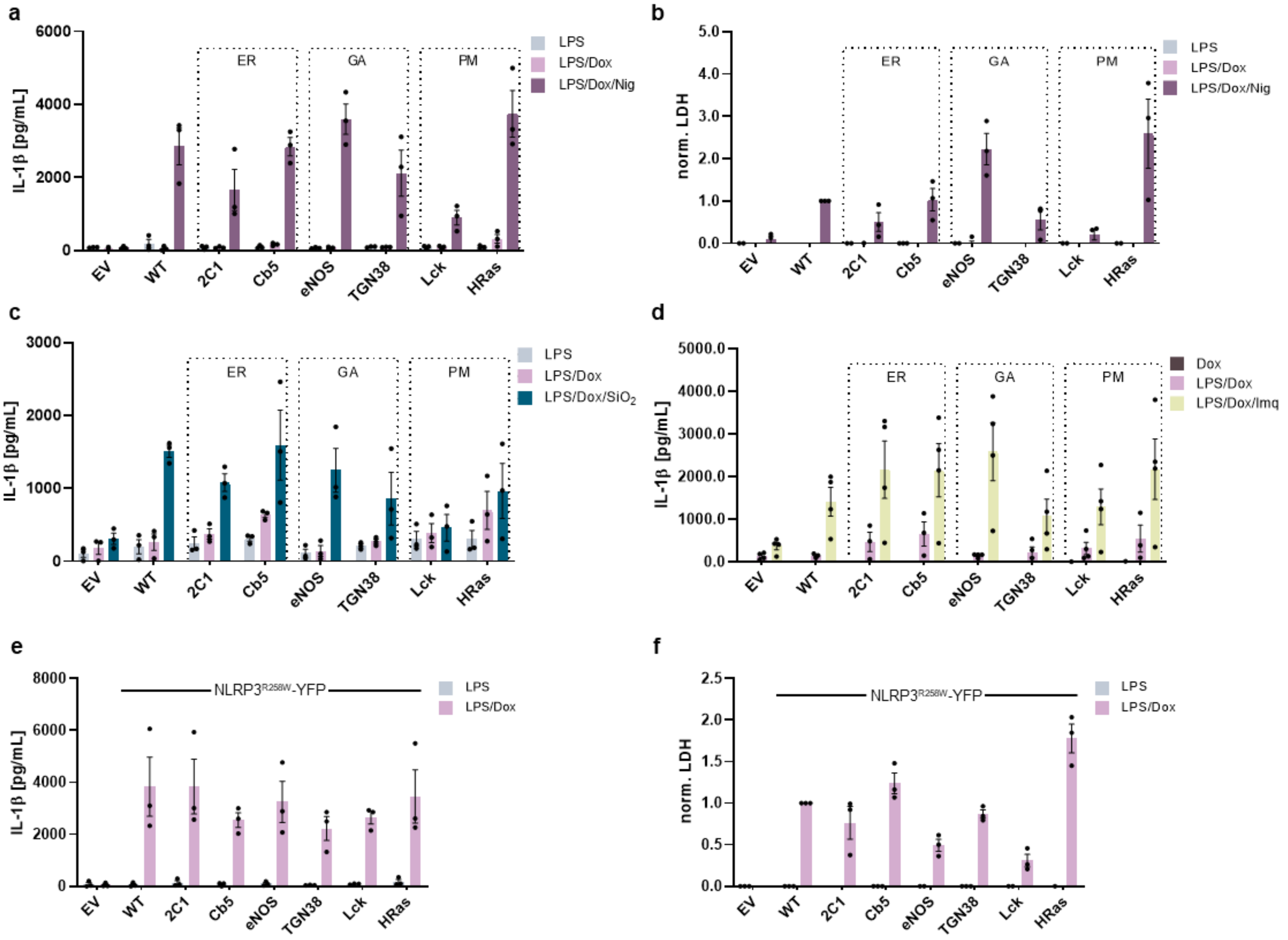

Supplementary Fig. 3. **Designed membrane-tethered NLRP3 variants respond to canonical triggers**. **a-d** NLRP3-KO iBMDMs expressing indicated NLRP3 (**a-d**) or NLRP3^{R258W} (**e-f**) variants were primed with 100 ng/ml LPS and 1 µg/ml doxycycline (Dox) for 12 hours. After the medium exchange, cells were treated with 10 µM nigericin (Nig) for 1 hour (**a, b**), 0.2 mg/ml SiO_2 (**c**) or 20 ug/ml imiquimod (Imq) (**d**) for 6 hours. Graphs from (**a, c-e**) correspond to normalized data from Fig. 1f-i. EV, empty vector; WT, non-localized wild-type NLRP3. The mean ± SEM of three (**a-c, e-f**) or four (**d**) independent experiments is shown. One biological replicate for EV and WT is identical as in Supplementary Fig. 5h (**d**) and 8d (**f**) as the experiments were performed at the same time.

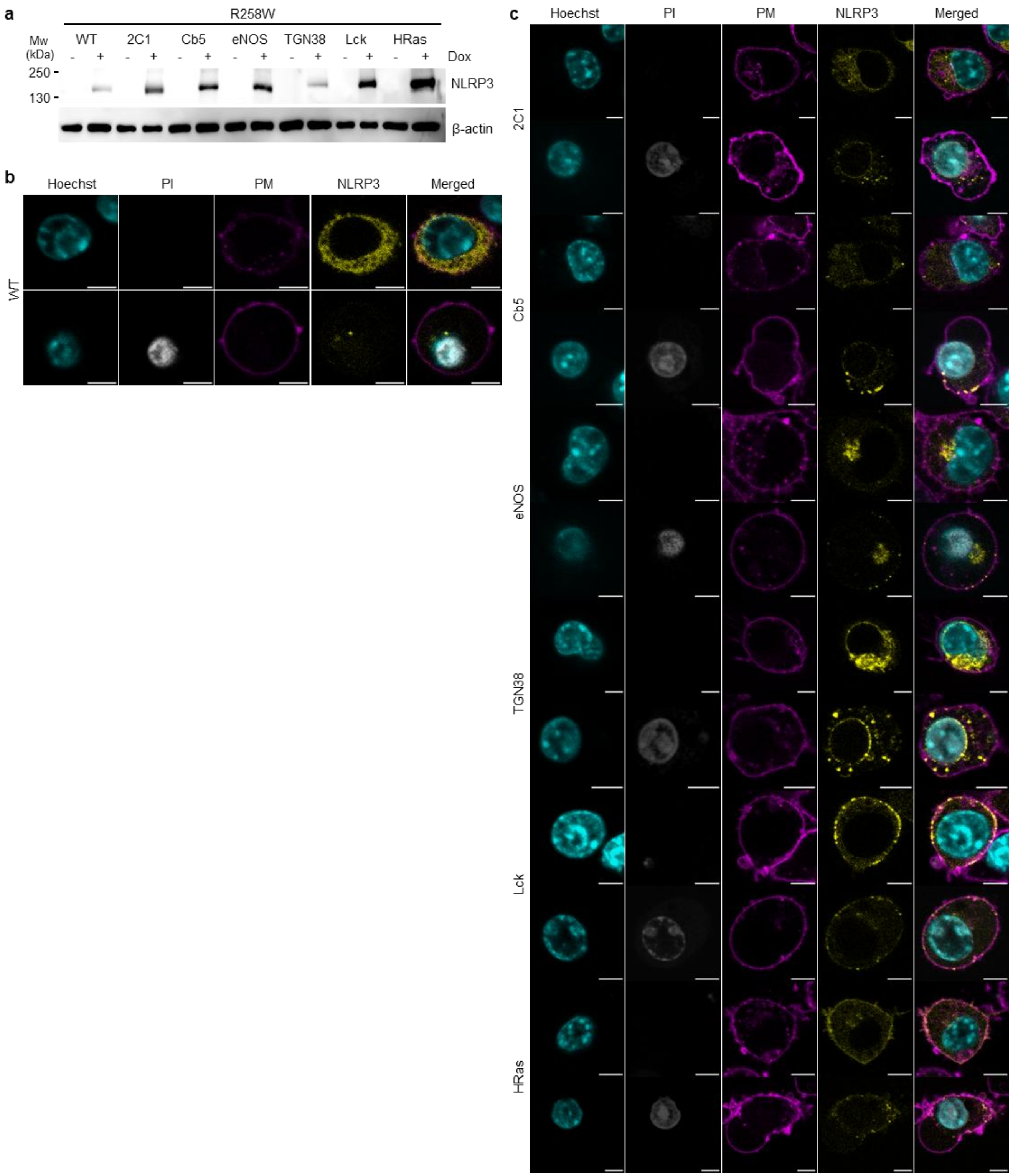

Supplementary Fig. 4. **Designed CAPS-related mutant organelle-enriched NLRP3 variants are constitutively active in NLRP3-KO iBMDMs. a** NLRP3-KO iBMDMs stably expressing indicated variants bearing R258W mutation within NLRP3 were primed with 100 ng/ml LPS and 1 μg/ml doxycycline (Dox) for 12 hours. Cells were lysed and immunoblotted against NLRP3. **b-c** NLRP3-KO iBMDMs stably expressing indicated wild-type (WT) (**b**) or organelle-enriched NLRP3^{R258W}-YFP variants (**c**) were primed as in (**a**). After 12 hours, nuclei were stained with Hoechst 33342 and plasma membrane (PM) with Cholera toxin subunit B. To follow membrane perforation, cells were stained with propidium iodide

(PI) prior to imaging. An example of PI-negative and PI-positive cell is shown for each NLRP3 variant. Scale bar, 5 µm. BF, brightfield. Western blots (**a**) and images (**b, c**) are representative of three independent experiments.

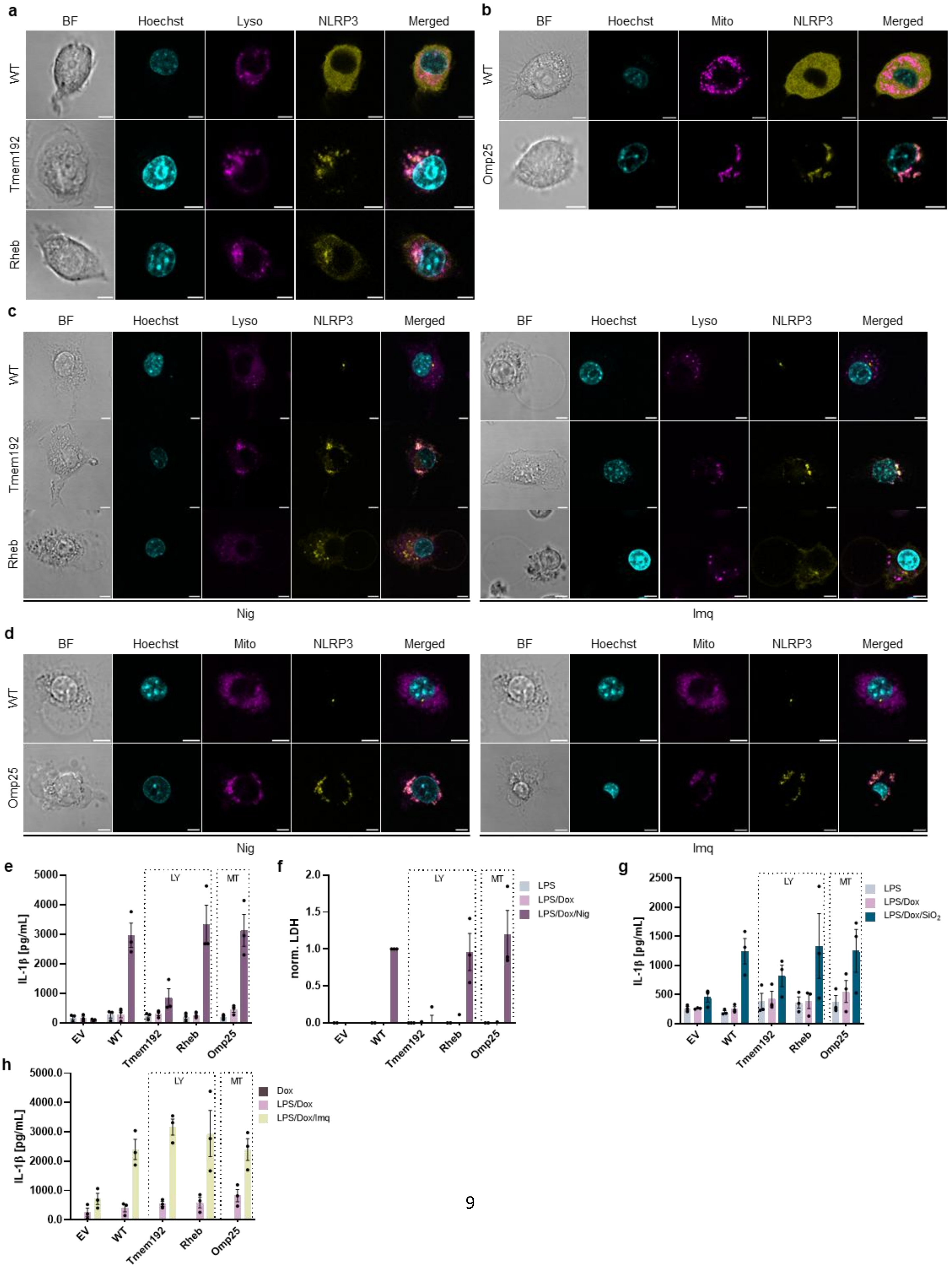
a
BF
Hoechst
Lyso
NLRP3
Merged
WT
Tmem192
Rheb
b
BF
Hoechst
Mito
NLRP3
Merged
WT
Omp25
c
BF
Hoechst
Lyso
NLRP3
Merged
WT
Tmem192
Rheb
Nig
Imq
d
BF
Hoechst
Mito
NLRP3
Merged
WT
Omp25
Nig
Imq
e
IL-1β [pg/mL]
LY
MT
EV
WT
Tmem192
Rheb
Omp25
f
norm. LDH
LY
MT
LPS
LPS/Dox
LPS/Dox/Nig
g
IL-1β [pg/mL]
LY
MT
LPS
LPS/Dox
LPS/Dox/SiO2
h
IL-1β [pg/mL]
LY
MT
Dox
LPS/Dox
LPS/Dox/Imq

Supplementary Fig. 5. **NLRP3 variants remain tethered to lysosomes or mitochondria despite nigericin or imiquimod treatment. a-d** NLRP3-KO iBMDMs stably expressing indicated wild-type (WT) or organelle-enriched NLRP3-YFP variants were primed with LPS in the presence of doxycycline. Post-priming, cells were first stained with LysoTracker Deep Red for lysosomes (Lyso) (**a, c**) or mitochondria (Mito) (**b, d**) with MitoTracker Deep Red followed by Hoechst 33342 staining for nuclei. After that, cells were activated with nigericin (Nig) or imiquimod (Imq) (**c-d**) and imaged within 1 or 1.5 hours, respectively. Scale bar, 5 µm. BF, brightfield. Images (**a-d**) are representative of three independent experiments. **e-h** Cells were primed as in (**a-d**) and treated with nigericin (**e-f**), SiO_2 (**g**) or imiquimod (**h**). Panels (**e-h**) represent the mean ± SEM of three independent experiments. Panels (**e, g-h**) correspond to non-normalized data from Fig. 2e-g. One set of data for EV and WT (**h**) is identical in Supplementary Fig. 3d, as the experiments were performed at the same time.

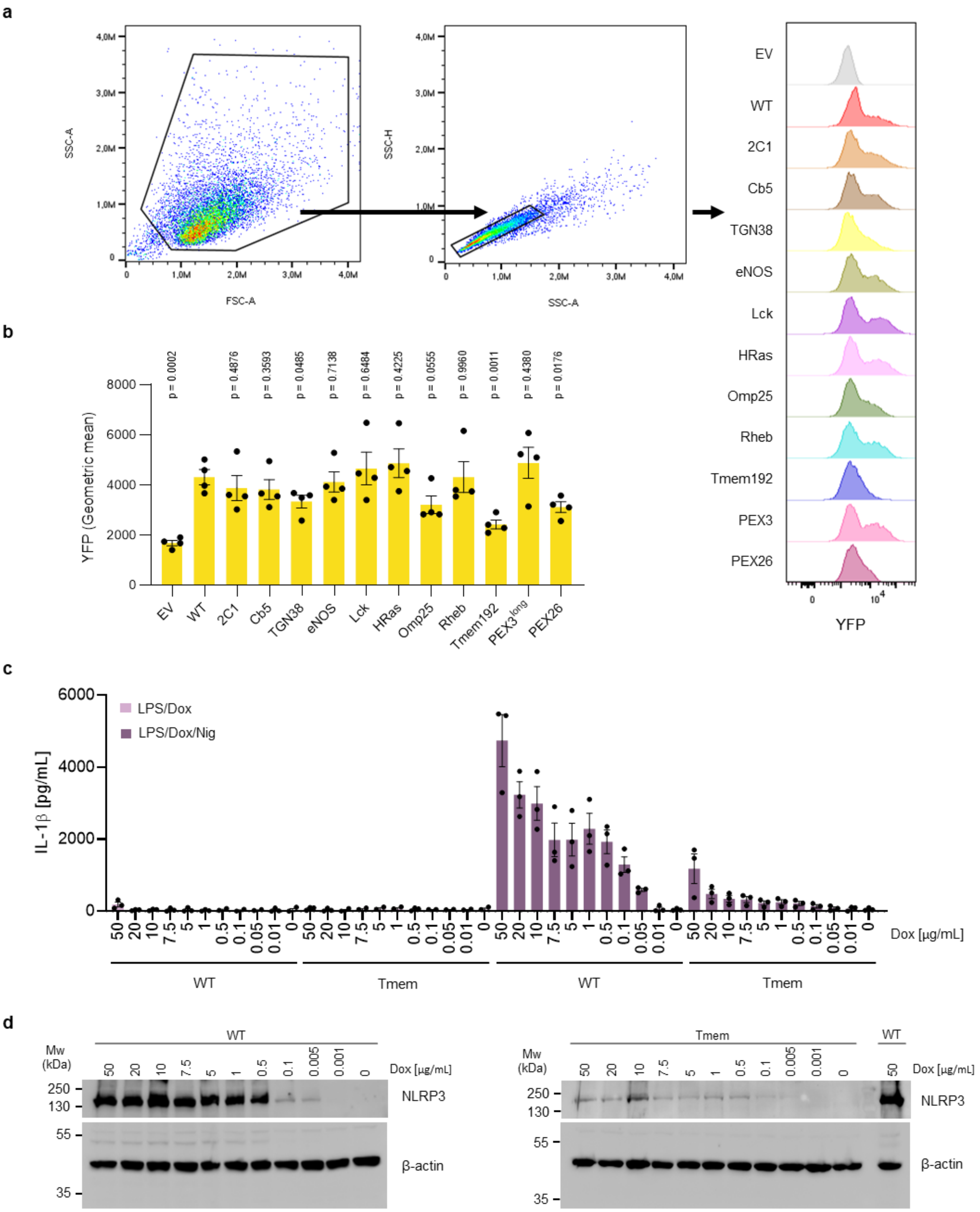

a
SSC-A
FSC-A
SSC-H
SSC-A
EV
WT
2C1
Cb5
TGN38
eNOS
Lck
HRas
Omp25
Rheb
Tmem192
PEX3
PEX26
YFP
b
YFP (Geometric mean)
p = 0.0002
p = 0.4876
p = 0.3593
p = 0.0485
p = 0.7138
p = 0.6484
p = 0.4225
p = 0.0555
p = 0.9960
p = 0.0011
p = 0.4380
p = 0.0176
EV
WT
2C1
Cb5
TGN38
eNOS
Lck
HRas
Omp25
Rheb
Tmem192
PEX3long
PEX26
c
IL-1β [pg/mL]
LPS/Dox
LPS/Dox/Nig
Dox [µg/mL]
WT
Tmem
WT
Tmem
d
Mw (kDa)
WT
Dox [µg/mL]
NLRP3
β-actin
Tmem
WT

Supplementary Fig. 6. **Expression levels of NLRP3-YFP variants**. **a-b** NLRP3-KO iBMDMs expressing indicated NLRP3-YFP variants were treated with LPS and doxycycline (Dox) overnight. Flow cytometry was used to assess YFP fluorescence (**b**) using the presented gating strategy (**a**). **c-d** Cells expressing WT or Tmem192 variant were stimulated with LPS and increasing concentrations of doxycycline. After 12 hours, medium was exchanged with nigericin (Nig) for 1 hour (**c**). Bars represent the mean ± SEM from three (**c**) or four (**b**) biological replicates. Western blots (**d**) are representative of four repeats. An unpaired two-tailed *t*-test was used to compare the differences between the expression levels of localized variants to non-localized NLRP3-YFP (WT).

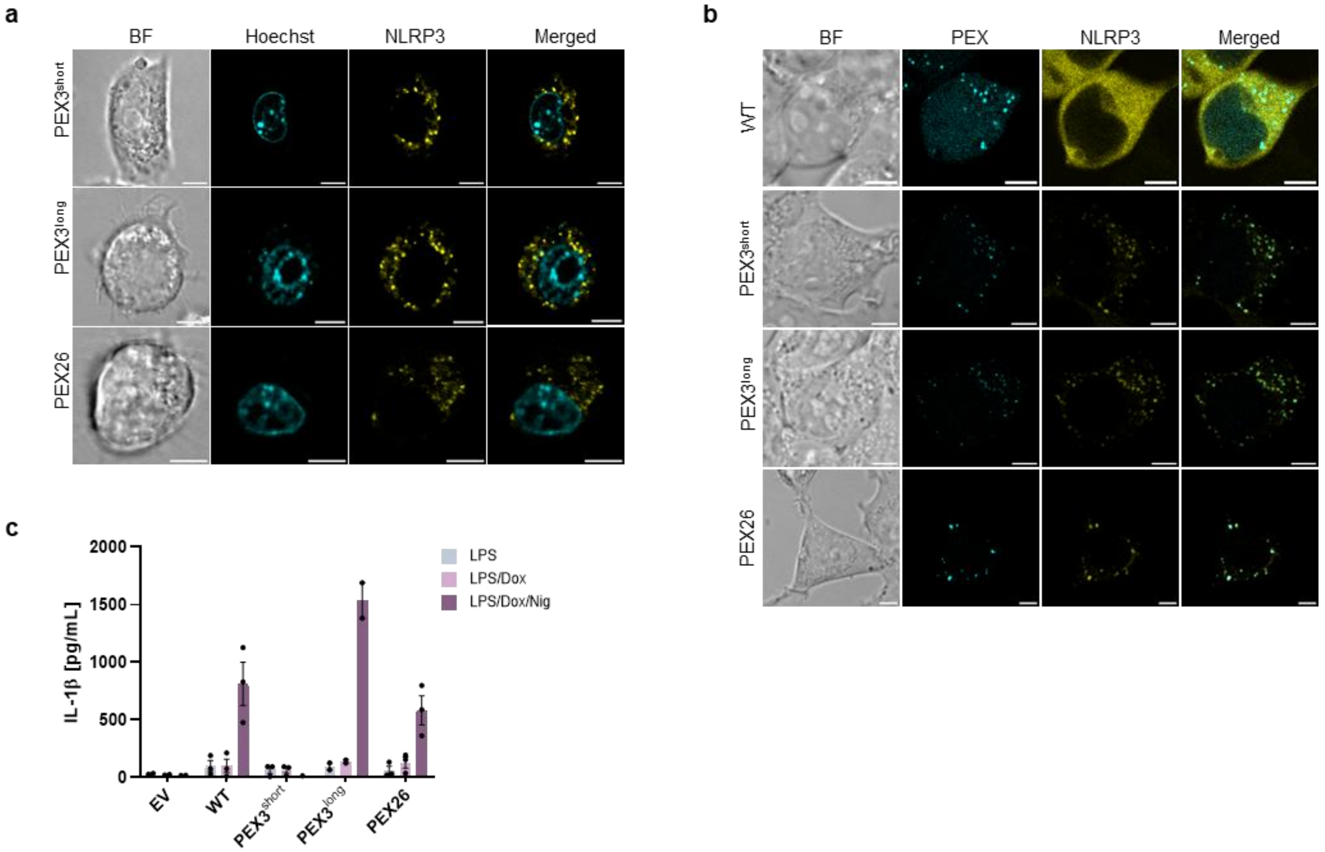

Supplementary Fig. 7. **NLRP3 can be activated on peroxisomal membrane. a** Peroxisome-restricted variants were primed with 100 ng/mL LPS and 1 µg/mL doxycycline (Dox) overnight and imaged after nuclear staining with Hoechst 33342. **b** HEK293T cells were transfected with respective NLRP3 construct and mCerulean-tagged protein marker for peroxisomes (PEX). Images were taken 18 hours post-transfection. Scale bar, 5 µm. BF, brightfield. Images are representative of at least three independent experiments. **c** iBMDMs were primed as in (**a**) and treated with nigericin (Nig). Non-normalized mean ± SEM of three independent experiments (except for $PEX3^{long}$, where N=2) from Fig. 2i is shown.

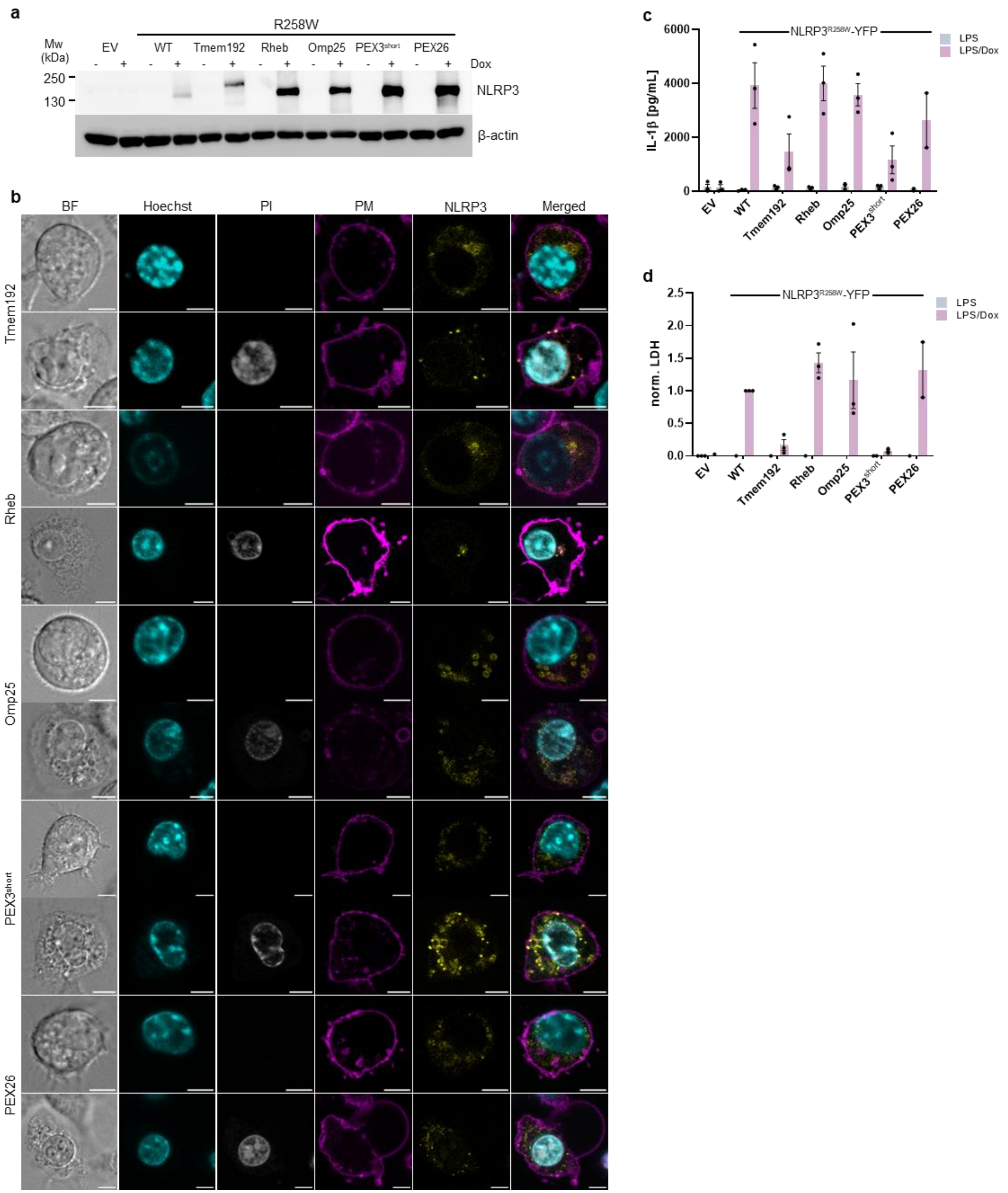

Supplementary Fig. 8. **R258W mutant NLRP3 variants at lysosomes, mitochondria and peroxisomes are constitutively active.** **a** NLRP3-KO iBMDMs stably transduced with indicated tags fused to NLRP3^{R258W}-YFP were primed with 100 ng/ml LPS and 1 µg/ml doxycycline (Dox) for 12 hours. **b** Cells were treated as in (**a**). Post-priming, cells were stained with Hoechst 33342 and Cholera toxin subunit B (PM). To discriminate between live and dead cells, propidium iodide (PI) was used prior to imaging. An example of PI-negative and PI-positive cell is shown for each NLRP3 variant. Scale bar, 5 µm. BF, brightfield. Data (**a, b**) is representative of three independent experiments. **c-d** Cells were primed as in (**a**). Data from (**c**) corresponds to normalized data from Fig. 2j. Bars represent the mean ± SEM of two (PEX26 in **c, d**) or three (**c, d**)

independent experiments. One biological repeat for EV and WT (**d**) is the same as in Supplementary Fig. 3f, as the experiments were performed at the same time.

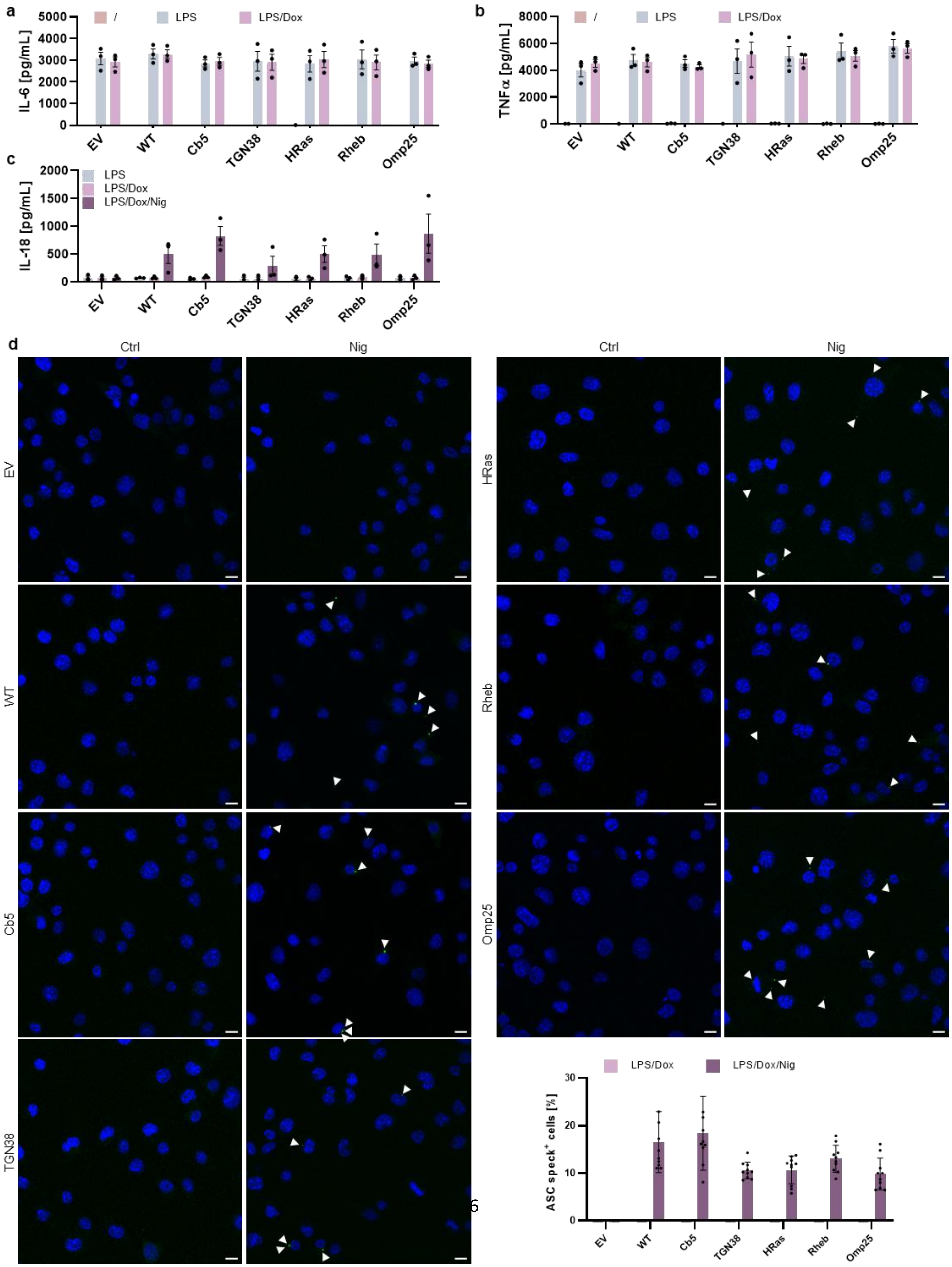
a
b
c
d
/
LPS
LPS/Dox
LPS/Dox/Nig
IL-6 [pg/mL]
TNFα [pg/mL]
IL-18 [pg/mL]
ASC speck⁺ cells [%]
EV
WT
Cb5
TGN38
HRas
Rheb
Omp25
Ctrl
Nig

Supplementary Fig. 9. **Organelle-tethered NLRP3 variants induce ASC speck formation upon nigericin treatment. a-c** NLRP3-KO iBMDMs were primed with LPS and doxycycline (Dox) or left untreated for 12 hours. The efficacy of priming was followed by the measurement of IL-6 (**a**) or TNFα (**b**) in cell supernatants. Post-priming, cells were treated with nigericin (Nig) (**c**). Graphs in (**a-b**) present non-normalized data corresponding to Fig. 3a-b. Data (**a-c**) represents the mean ± SEM of three independent experiments. **d** After priming as in (**a-c**), cells were stimulated with nigericin for 20 minutes or left untreated (Ctrl), afterwards ASC specks (green) were detected by immunofluorescent staining. Nuclei were stained with DAPI (blue). White arrows indicate individual ASC specks. 5 randomly selected frames from two independent experiments were used for the quantification of ASC speck-positive cells. Scale bar, 10 μm.

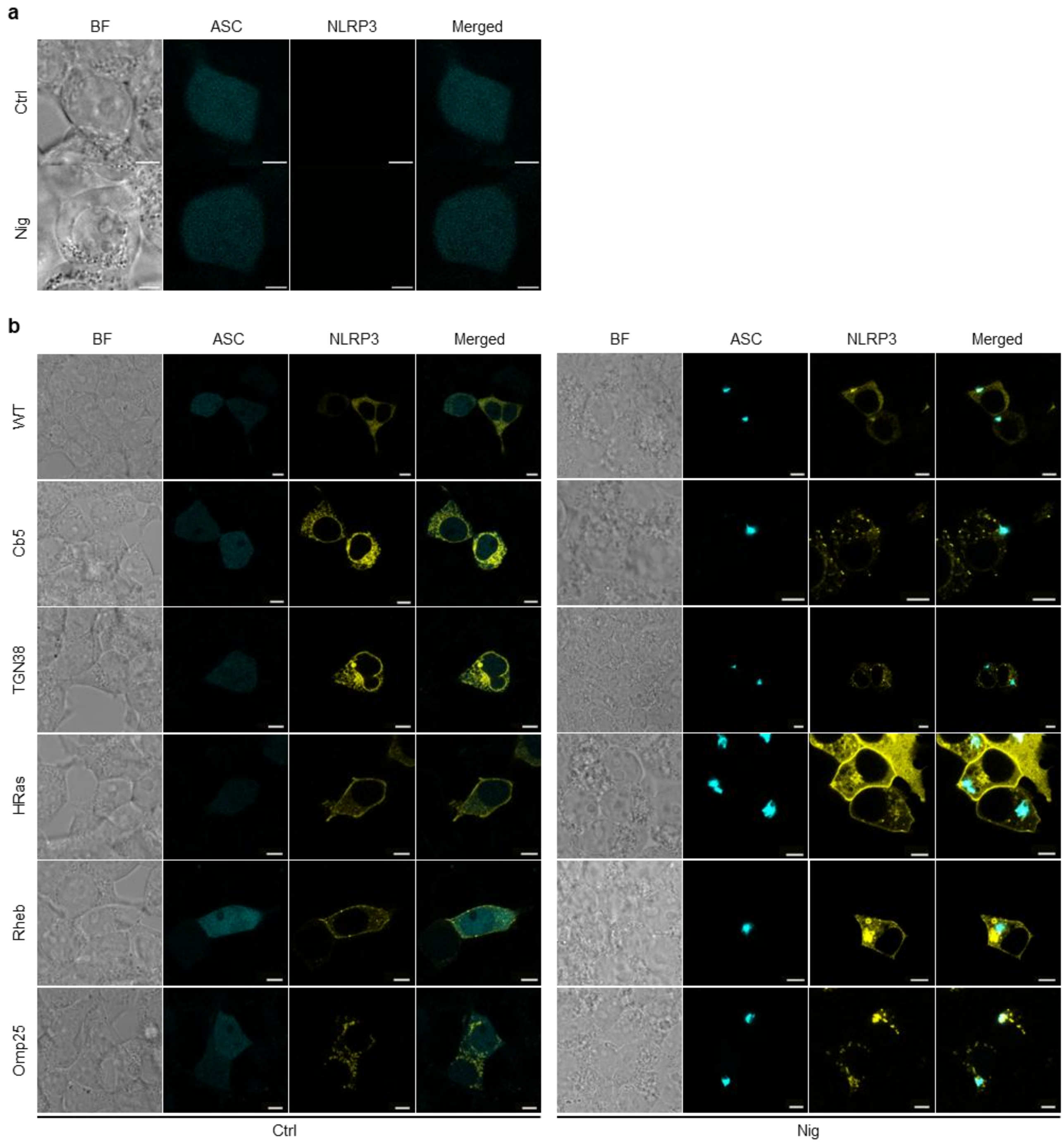

Supplementary Fig. 10. **Organelle-enriched NLRP3 variants support ASC speck formation. a-b** HEK293T cells were transfected with ASC-mCerulean (cyan) without plasmid encoding NLRP3 (**a**) or with ASC-mCerulean and indicated NLRP3-YFP variant (**b**). Cells were visualized 17 hours post-transfection with or without nigericin (Nig) treatment. Scale bar, 5 µm. BF, brightfield. Representative images from three independent experiments are shown.

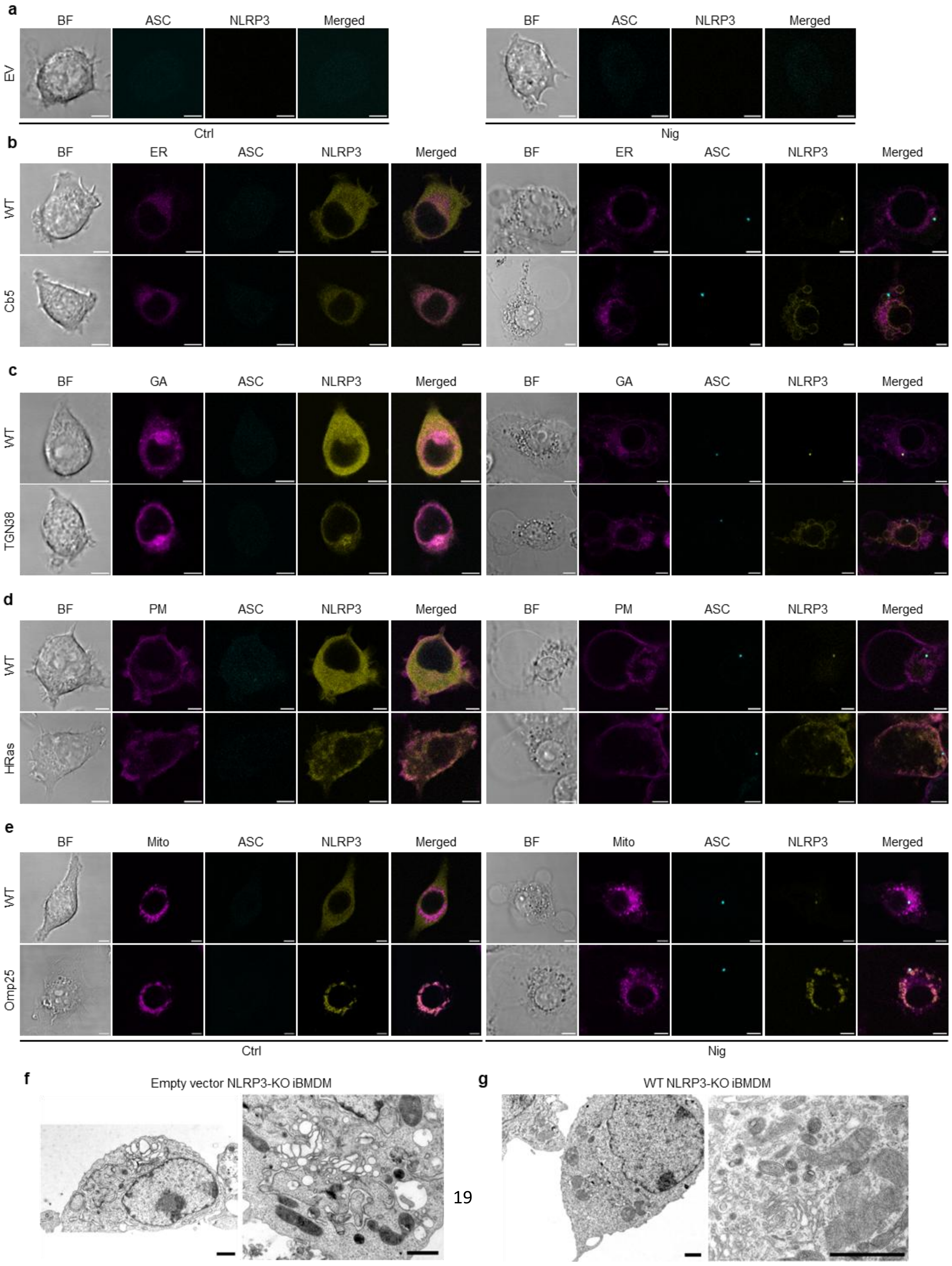
a
BF
ASC
NLRP3
Merged
EV
Ctrl
Nig
b
ER
WT
Cb5
c
GA
TGN38
d
PM
HRas
e
Mito
Omp25
f
Empty vector NLRP3-KO iBMDM
g
WT NLRP3-KO iBMDM

Supplementary Fig. 11. **Nigericin induces the formation of ASC specks proximally to membrane-tethered NLRP3 and organelles.** **a-e** iBMDMs transduced with empty vector control (EV) (**a**) or stably expressing indicated NLRP3-YFP variants (**b-e**) and ASC-mCerulean were stimulated with doxycycline for 12 hours following 4-hour activation with 1 µg/mL LPS. After 30–40-minute staining of endoplasmic reticulum (ER) with ER-Tracker Red (**b**), Golgi apparatus (GA) with BODIPY TR C_5 (**c**), plasma membrane (PM) with Cholera toxin subunit B, Alexa Fluor 647 (**d**) or mitochondria (Mito) with MitoTracker Deep Red, cells were treated with nigericin (Nig) or left untreated (Ctrl) and imaged within 1-hour post-stimulation. Scale bar, 5 µm. **f-g** LPS and doxycycline-primed cells were treated with nigericin (**f**) or left untreated (**g**). Treatment with nigericin resulted in ultrastructural changes, such as increased GA (**f**). Scale bar, 1 µm. Microscopic images (**a-g**) are representative of three independent experiments.

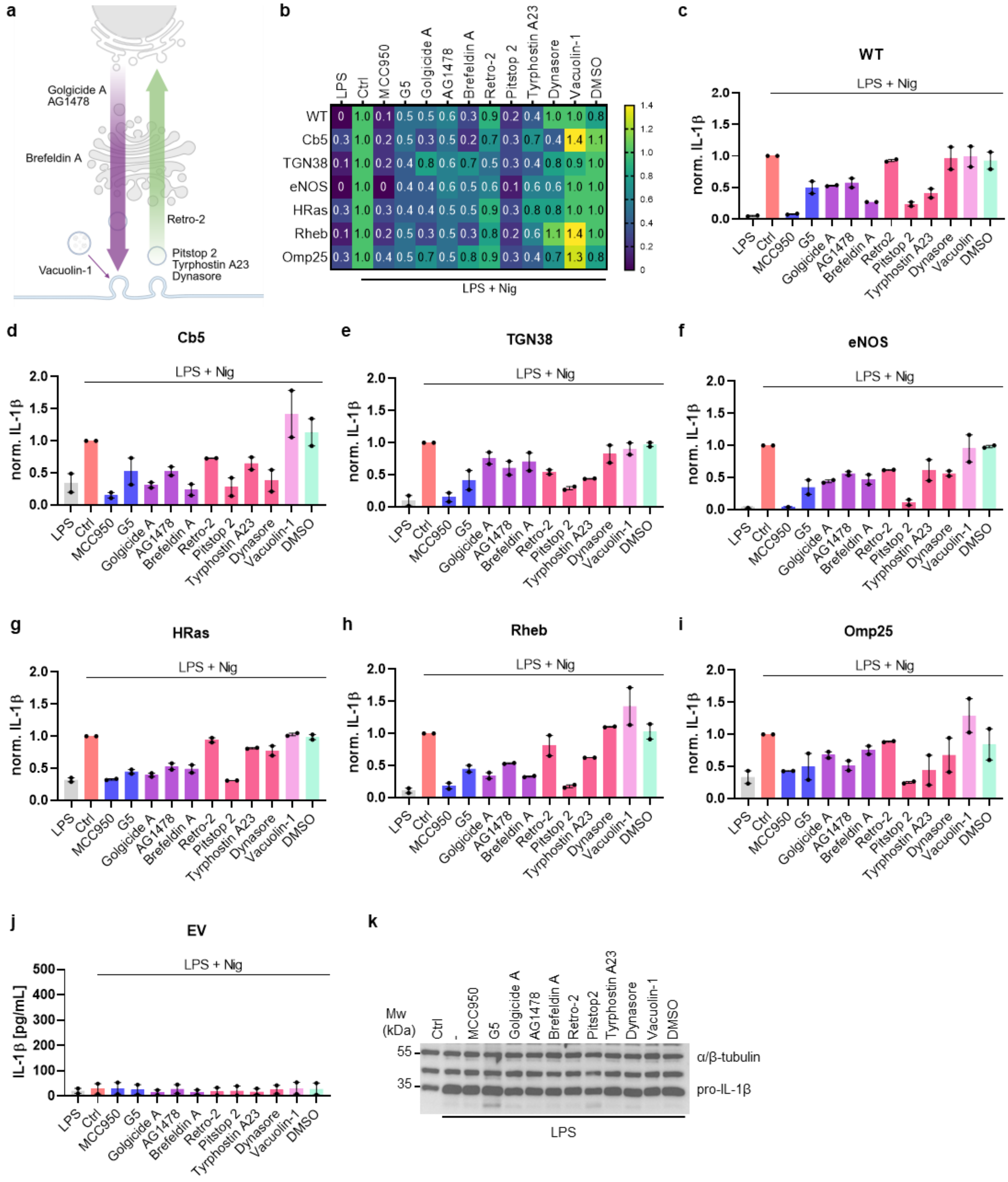

Supplementary Fig. 12. **Anterograde and retrograde trafficking inhibitors fail to define a preferred subcellular site for NLRP3 activation. a** Stages of cellular transport targeted by inhibitors. **b-j** Cells were primed with LPS and doxycycline overnight. Indicated inhibitors or DMSO were added 4 hours before nigericin (Nig). Values within the heatmap (**b**) or on graphs (**c-i**) show fold change of IL-1β release relative to respective LPS- and nigericin-treated cell lines, except for empty vector (EV) transduced cells (**j**). **k** NLRP3-KO iBMDMs expressing non-localized wild-type NLRP3 (WT) were primed as in (**b-j**) following treatment with specified compounds as in (**b-j**). The effect of inhibitors on priming was evaluated with expression of pro-IL-1β. Data (**b-j**) are mean ± SEM of two

independent experiments. Western blot (**k**) is representative of two independent experiments. Image (**a**) created in Biorender. Hafner Bratkovic, I. (2025) https://BioRender.com/rn6lehg.

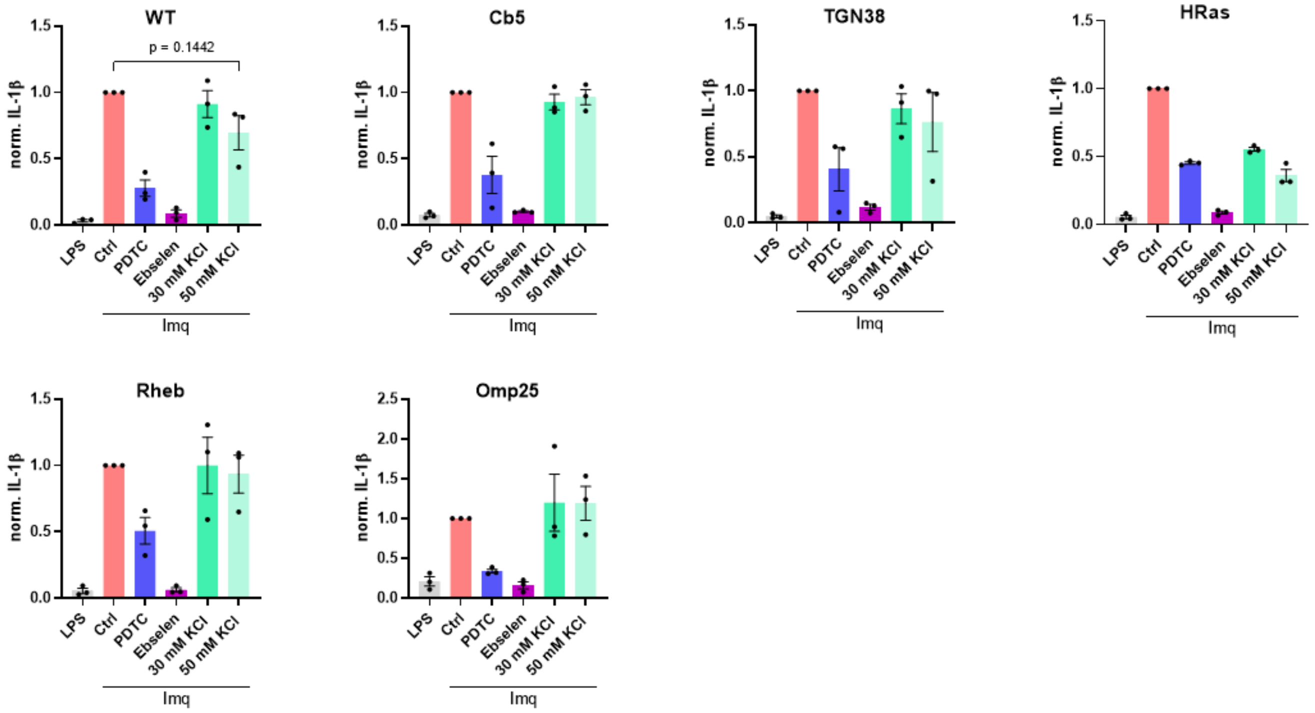

Supplementary Fig. 13. **The effect of KCl and ROS inhibitors on inflammasome stimulation by imiquimod.** After priming, the medium was exchanged for fresh medium containing KCl or ROS inhibitors and 30 minutes later, imiquimod was added for 1 hour, after which the supernatants were analyzed with IL-1β ELISA. Data are mean ± SEM of N=3 independent experiments. Two-tailed unpaired *t*-test with Welch correction was used for the comparison of populations.

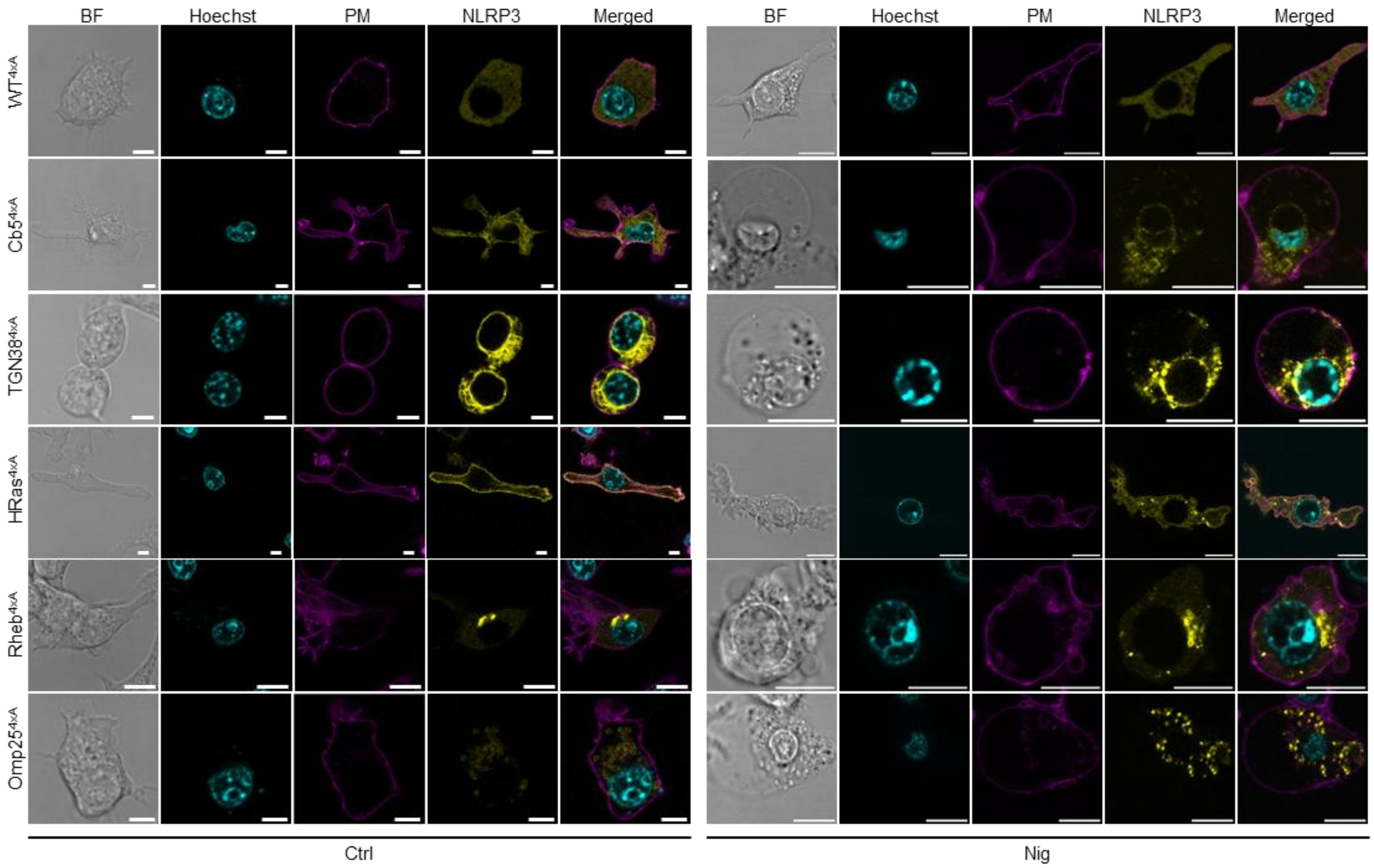

Supplementary Fig. 14. **Organelle-restricted NLRP3 variants with mutated charged segment facilitate inflammasome assembly.** NLRP3-KO iBMDMs stably expressing indicated wild-type (WT) or organelle-enriched NLRP3-YFP with mutated polybasic segment (4xA) were primed with 100 ng/ml LPS and 1 μg/ml doxycycline. After 12 hours, cells were treated with nigericin (Nig) or left untreated (Ctrl) and stained with Hoechst 33342 for nuclei and with Cholera toxin subunit B for plasma membrane (PM). Scale bar, 10 μm. BF, brightfield. Images are representative of three independent experiments.

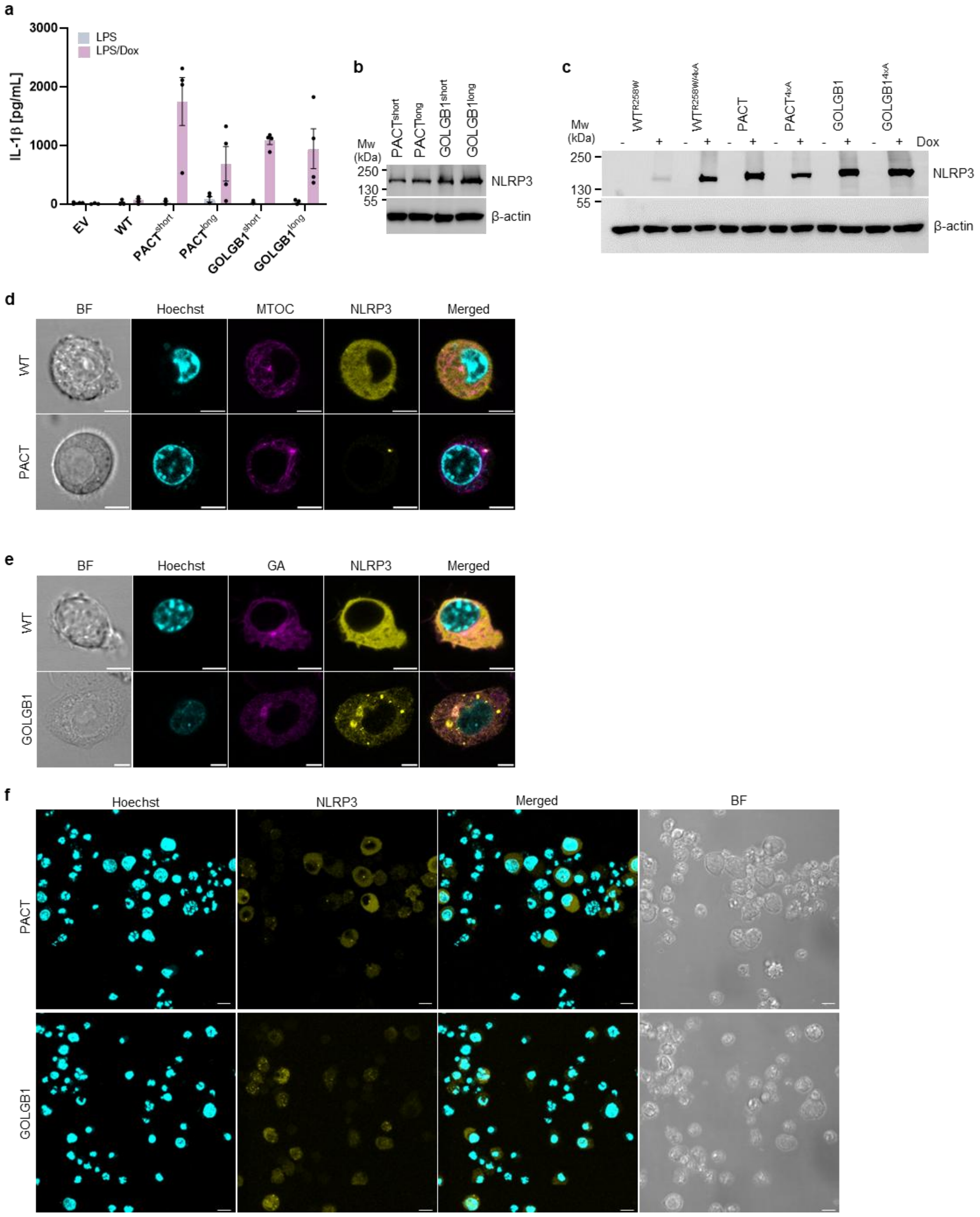
a
3000
2000
1000
0
IL-1β [pg/mL]
LPS
LPS/Dox
EV
WT
PACTshort
PACTlong
GOLGB1short
GOLGB1long
b
PACTshort
PACTlong
GOLGB1short
GOLGB1long
Mw (kDa)
250
130
55
NLRP3
β-actin
c
WTR258W
WTR258W/4xA
PACT
PACT4xA
GOLGB1
GOLGB14xA
Mw (kDa)
250
130
55
Dox
NLRP3
β-actin
d
BF
Hoechst
MTOC
NLRP3
Merged
WT
PACT
e
BF
Hoechst
GA
NLRP3
Merged
WT
GOLGB1
f
Hoechst
NLRP3
Merged
BF
PACT
GOLGB1

Supplementary Fig. 15. **Clustering of NLRP3 leads to inflammasome activation. a-b** NLRP3-KO iBMDMs expressing variants with either short (6 amino acids) or long (20 amino acids) linker between the tag and NLRP3 were primed with LPS and doxycycline (Dox) overnight. Cells were subsequently stimulated with 10 μM nigericin (Nig) for 1 hour (**a**), and IL-1β levels were measured. Data are presented as mean ± SEM from four independent experiments. **c** Representative immunoblot of WT, PACT and GOLGB1 expressing iBMDMs with respective NLRP3$^{K127A/K128A/K129A/K130A}$ (4xA) mutants. **d-f** Wild-type (WT), centrosome-tagged (PACT) or Golgi-tagged (GOLGB1) NLRP3-KO iBMDM were primed as in (**a-b**). Following Hoechst 33342 staining, cells were imaged for determination of NLRP3 puncta (**f**) or further stained with γ-tubulin to determine the localization of centrosome (**d**) or with BODIPY TR C_5 for Golgi apparatus (**e**). Scale bar, 5 μm (**d, e**) or 10 μm (**f**). Immunoblots (**b, c**) and images (**d-f**) are representative of three independent experiments. BF, brightfield.

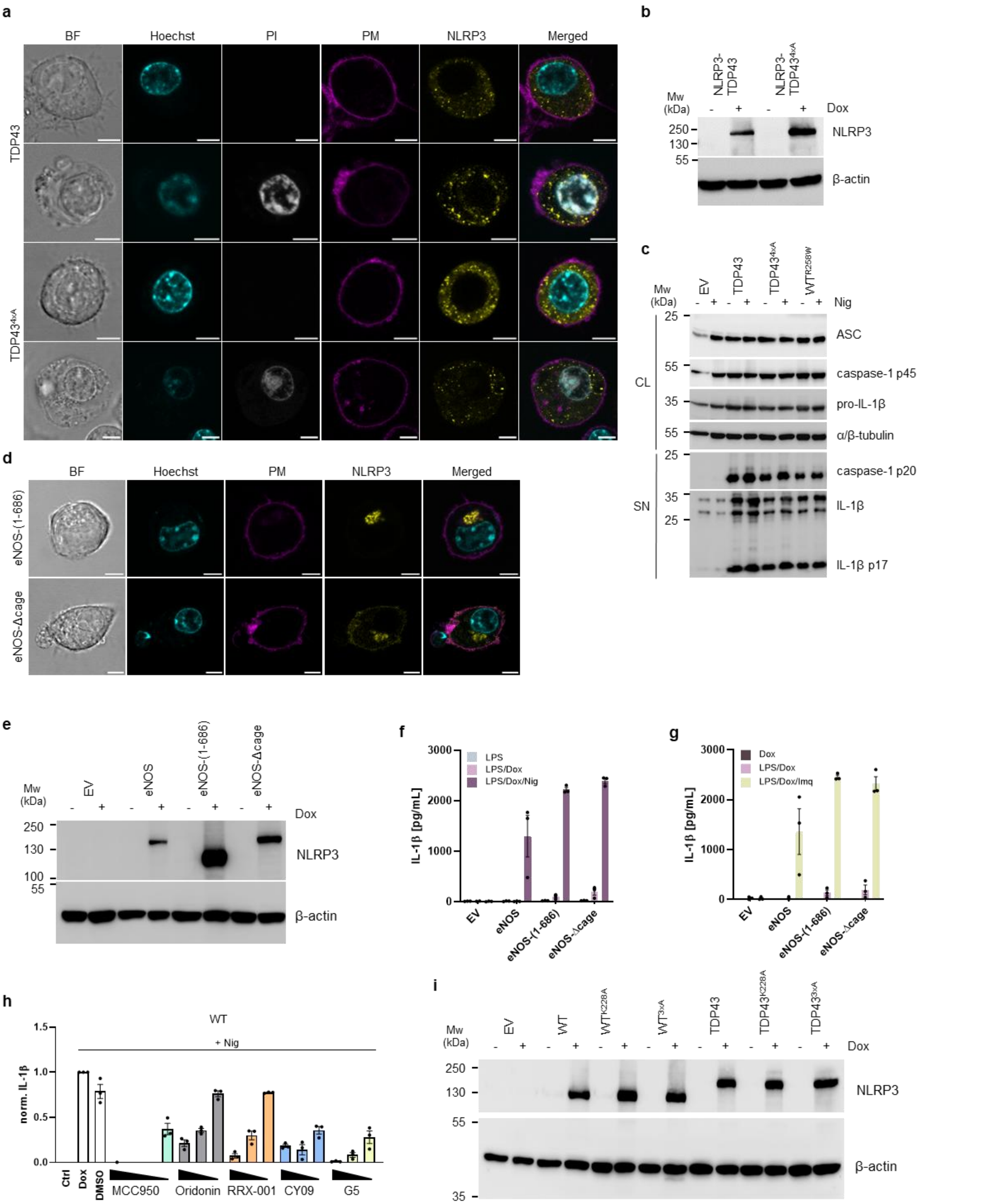

Supplementary Fig. 16. **TDP43 serves as a protein scaffold to cluster NLRP3, but not due to inactive cage disassembly. a** NLRP3-KO iBMDMs stably transduced with TDP43 NLRP3 or TDP43 NLRP3$^{K127A/K128A/K129A/K130A}$ (4xA) mutant were stained with propidium

iodide (PI), Hoechst 33342 and Cholera toxin subunit B (PM) after 12 hours of priming with 100 ng/ml LPS and 1 µg/ml doxycycline (Dox). Scale bar, 5 µm. **b-c** Cells were primed as in (**a**) and further treated with nigericin (**c**) after which cell lysates (CL) and supernatants were blotted against inflammasome components (**c**). **d** NLRP3-KO iBMDMs expressing eNOS variants were treated as in (**a**) and stained with Hoechst 33342 for nuclei and with Cholera toxin subunit B for plasma membrane (PM). Scale bar, 5 µm. **e-g** eNOS variant cells were primed as in (**a**) and further treated with nigericin (Nig) for 1 hour (**f**) or imiquimod (Imq) for 6 hours (**g**). **h** NLRP3-KO iBMDMs expressing WT variant were stimulated with LPS and Dox after 30-minute pre-incubation with inhibitors or DMSO, except for Ctrl, where only LPS was added. After 12-hour incubation, nigericin was added for 1 hour. Measurements of IL-1β were normalized to LPS/Dox treated cells. **i** Representative western blot of NLRP3 Walker A mutants K228A or G227A/K228A/T229A (3xA) or corresponding TDP43 fusion proteins in primed iBMDM cells. BF, brightfield. Data (**f, g, h**) are mean ± SEM of three independent experiments. Images (**a, d**) and western blots (**b, c, e, i**) are representative of three independent experiments.

Supplementary Table 1. **Origins of localization tags used for NLRP3 enrichment at organelles.** Murine NLRP3 containing a yellow fluorescent protein tag was fused to localization sequences of indicated organelle-specific proteins via N- or C-termini. Different mechanisms of localization to the membrane were used, either lipidation motifs or transmembrane helices which are underlined.

LOCALIZATION	TAG SOURCE	AMINO ACID SEQUENCE	TYPE
**Endoplasmic reticulum**	2C1(1-27)	MDPVVVLGLCLSCLLLLSLWKQSYGGG	Transmembrane
	Cb5(100-134)	ITTVESNSSWWTNWVIPAISALVVALMYRLYMAED	Transmembrane
**Golgi apparatus**	eNOS(1-32)	MGNLKSVAQEPGPPCGLGLGLGLGLCGKQGPA	Lipidation (myristoylation, palmitoylation)
	TGN38(304-357)	HFFAYLVTAAVLVAVLYIAYHNKRKIIAFALEGKRSKVTRRPKASDY<br>QRLNLKL	Transmembrane
	GOLGB1(3131-3259)	EPQQSFSEAQQQLCNTRQEVNELRKLLEEERDQRVAAENALSVAEEQ<br>IRRLEHSEWDSSRTPIIGSCGTQEQALLIDLTSNSCRRTRSGVGWKR<br>VLRSLCHSRTRVPLLAAIYFLMIHVLLILCFTGHL	Transmembrane
**Plasma membrane**	Lck(1-12)	MGCWCSSNPEDD	Lipidation (myristoylation, palmitoylation)
	HRas(165-189)	QHKLRKLNPPDESGPGCMSCKCVLS	Lipidation (palmitoylation, farnesylation)
**Lysosome**	Tmem192	MAAGGRMEDGSLDITQSIEDDPLLDAQLLPHHSLQAHFRPRFHPLPT<br>VIIVNLLWFIHLVFVVLAFLTGVLCSYPNPNEDKCPGNYTNPLKVQT<br>VIILGKVILWILHLLLECYIQYHHSKIRNRGYNLIYRSTRHLKRLAL<br>MIQSSGNTVLLLILCMQHSFPEPGRLYLDLILAILALELICSLICLL<br>IYTVKIRRFNKAKPEPDILEEEKIYAYPSNITSETGFRTISSLEEIV<br>EKQGDTIEYLKRHNALLSKRLLALTSSDLGCQPSRT	Transmembrane
	Rheb(170-184)	MDGAASQGKSSCSVM	Lipidation (farnesylation)
**Mitochondria**	Omp25(198-145)	VHRGDGEPSGVPVAVVLLPVFALTLVAVWAFVRYRKQL	Transmembrane
**Peroxisome**	PEX3(1-42)	MLRSVWNFLKRHKKKCIFLGTVLGGVYILGKYGQKKIREIQE	Transmembrane
	PEX26(237-305)	RQLWDSAVSHFFSLPFKKSLLAALILCLLVVRFDPASPSSLHFLYKL<br>AQLFRWIRKAAFSRLYQLRIRD	Transmembrane
**Microtubule organizing center**	PACT(3699–3790)	KRIYGKYLRAESFRKALIYQKKYLLLLLGGFQECEDATLALLARMGG<br>QPAFTDLEVITNRPKGFTRFRSAVRVSIAISRMKFLVRRWHRVTG	/

Supplementary Table 2. **Design of organelle-enriched NLRP3 variants.** Murine NLRP3 (black) was fused to a yellow fluorescence protein (YFP) (yellow) using a linker sequence in red. Organelle-enriched NLRP3 variants were prepared by the addition of a designated localization tag (green) separated by the linker (magenta) to N- or C-termini of NLRP3 in a way that NLRP3 resides at the cytosolic face of the organelles.

Construct	Sequence
2C1-linker-NLRP3-linker-YFP	MDPVVVLGLCLSCLLLLSLWKQSYGGGSGPGSGTSVRCKLAQYLEDLEDVDLKKFKMHLEDYPPEKGCIPVPRGQMEKADH<br>LDLATLMIDFNGEEKAWAMAVWIFAAINRRDLWEKAKKDQPEWNDTCTSHSSMVCQEDSLEEEWMGLLGYLSRISICKKKK<br>DYCKMYRRHVRSRFYSIKDRNARLGESVDLNSRYTQLQLVKEHPSKQEREHELLTIGRTKMRDSPMSSLKLELLFEPEDGH<br>SEPVHTVVFQGAAGIGKTILARKIMLDWALGKLFKDKFDYLFFIHCREVSLRTPRSLADLIVSCWPDPNPPVCKILRKPSR<br>ILFLMDGFDELQGAFDEHIGEVCTDWQKAVRGDILLSSLIRKKLLPKASLLITTRPVALEKLQHLLDHPRHVEILGFSEAK<br>RKEYFFKYFSNELQAREAFRLIQENEVLFTMCFIPLVCWIVCTGLKQQMETGKSLAQTSKTTTAVYVFFLSSLLQSRGGIE<br>EHLFSDYLQGLCSLAADGIWNQKILFEECDLRKHGLQKTDVSAFLRMNVFQKEVDCERFYSFSHMTFQEFFAAMYYLLEEE<br>AEGETVRKGPGGCSDLLNRDVKVLLENYGKFEKGYLIFVVRFLFGLVNQERTSYLEKKLSCKISQQVRLELLKWIEVKAKA<br>KKLQWQPSQLELFYCLYEMQEEDFVQSAMDHFPKIEINLSTRMDHVVSSFCIKNCHRVKTLSLGFFHNSPKEEEEERRGGR<br>PLDQVQCVFPDTHVACSSRLVNCCLTSSFCRGLFSSLSTNRSLTELDLSDNTLGDPGMRVLCEALQHPGCNIQRLWLGRCG<br>LSHQCCFDISSVLSSSQKLVELDLSDNALGDFGIRLLCVGLKHLLCNLQKLWLVSCCLTSACCQDLALVLSSNHSLTRLYI<br>GENALGDSGVQVLCEKMKDPQCNLQKLGLVNSGLTSICCSALTSVLKTNQNFTHLYLRSNALGDTGLRLLCEGLLHPDCKL<br>QMLELDNCSLTSHSCWNLSTILTHNHSLRKLNLGNNDLGDLCVVTLCEVLKQQGCLLQSLQLGEMYLNRETKRALEALQEE<br>KPELTIVFEISWGGSGGVSKGEELFTGVVPILVELDGDVNGHKFSVSGEGEGDATYGKLTLKFICTTGKLPVPWPTLVTTF<br>GYGLQCFARYPDHMKQHDFFKSAMPEGYVQERTIFFKDDGNYKTRAEVKFEGDTLVNRIELKGIDFKEDGNILGHKLEYNY<br>NSHNVYIMADKQKNGIKVNFKIRHNIEDGSVQLADHYQQNTPIGDGPVLLPDNHYLSYQSALSKDPNEKRDHMVLLEFVTA<br>AGITLGMDELYKR
NLRP3-linker-YFP -linker- Cb5	MTSVRCKLAQYLEDLEDVDLKKFKMHLEDYPPEKGCIPVPRGQMEKADHLDLATLMIDFNGEEKAWAMAVWIFAAINRRDL<br>WEKAKKDQPEWNDTCTSHSSMVCQEDSLEEEWMGLLGYLSRISICKKKKDYCKMYRRHVRSRFYSIKDRNARLGESVDLNS<br>RYTQLQLVKEHPSKQEREHELLTIGRTKMRDSPMSSLKLELLFEPEDGHSEPVHTVVFQGAAGIGKTILARKIMLDWALGK<br>LFKDKFDYLFFIHCREVSLRTPRSLADLIVSCWPDPNPPVCKILRKPSRILFLMDGFDELQGAFDEHIGEVCTDWQKAVRG<br>DILLSSLIRKKLLPKASLLITTRPVALEKLQHLLDHPRHVEILGFSEAKRKEYFFKYFSNELQAREAFRLIQENEVLFTMC<br>FIPLVCWIVCTGLKQQMETGKSLAQTSKTTTAVYVFFLSSLLQSRGGIEEHLFSDYLQGLCSLAADGIWNQKILFEECDLR<br>KHGLQKTDVSAFLRMNVFQKEVDCERFYSFSHMTFQEFFAAMYYLLEEEAEGETVRKGPGGCSDLLNRDVKVLLENYGKFE<br>KGYLIFVVRFLFGLVNQERTSYLEKKLSCKISQQVRLELLKWIEVKAKAKKLQWQPSQLELFYCLYEMQEEDFVQSAMDHF<br>PKIEINLSTRMDHVVSSFCIKNCHRVKTLSLGFFHNSPKEEEEERRGGRPLDQVQCVFPDTHVACSSRLVNCCLTSSFCRG<br>LFSSLSTNRSLTELDLSDNTLGDPGMRVLCEALQHPGCNIQRLWLGRCGLSHQCCFDISSVLSSSQKLVELDLSDNALGDF<br>GIRLLCVGLKHLLCNLQKLWLVSCCLTSACCQDLALVLSSNHSLTRLYIGENALGDSGVQVLCEKMKDPQCNLQKLGLVNS<br>GLTSICCSALTSVLKTNQNFTHLYLRSNALGDTGLRLLCEGLLHPDCKLQMLELDNCSLTSHSCWNLSTILTHNHSLRKLN<br>LGNNDLGDLCVVTLCEVLKQQGCLLQSLQLGEMYLNRETKRALEALQEEKPELTIVFEISWGGSGGVSKGEELFTGVVPIL<br>VELDGDVNGHKFSVSGEGEGDATYGKLTLKFICTTGKLPVPWPTLVTTFGYGLQCFARYPDHMKQHDFFKSAMPEGYVQER<br>TIFFKDDGNYKTRAEVKFEGDTLVNRIELKGIDFKEDGNILGHKLEYNYNSHNVYIMADKQKNGIKVNFKIRHNIEDGSVQ<br>LADHYQQNTPIGDGPVLLPDNHYLSYQSALSKDPNEKRDHMVLLEFVTAAGITLGMDELYKRSGPGSGITTVESNSSWWTN<br>WVIPAISALVVALMYRLYMAED

Supplementary Table 3. **Oligonucleotide sequences.**

Oligonucleotide	Sequence
F-BamHI-NLRP3	CGGGATCCGCCACCATGAC
R-YFP	GGAATTCCTCATCTCTTGTACAGCTCGTCCATG
F-Tre3Gbackbone	CACAACACTTTTGTCTTATACTTGGATCC
R-Tre3Gbackbone	AGCGCCTCCCCTACCC
F-2C1	TACGGAGGTGGTTCAGGTCCCGGCAGTGGGACCTCCGTGCGGTGC
R-2C2	CCGAGTACCACGACTGGGTCCATGGTGGCGGATCCCAAGTATAAGACAAAAGTGTTGTGG
R-Cb5-NLRP3	ACGGTGGTGATGCCGCTGCCGGGG
F-NLRP3-Cb5	AGCGGCATCACCACCGTGGAGTCCA
R-Cb5	GGAATTCCTCAGTCCTCGGCCATGTACAGG
F-eNOS-NLRP3	CGGGATCCGCCACCATGGGAAACTTGAAGTCTGTTGCCCAAGAACCCGGCCCACCTTGCGGTCTTGGTCTTGGAC TTGGCTTGGGTCTGTGTGGAAAGC
F-TGN38	CGGGATCCGCCACCATGTCCGGACTCAGATCTCGAGC
R-NLRP3-TGN38	CCGGGGCCGCTAAGCTTTAGGTTCAAACGTTGGTAG
F-TGN38-NLRP3	ACCTAAAGCTTAGCGGCCCCGGC
R-NotI-YFP	ATTTGCGGCCGCTCATCTCTTGTACAGCTCGTCC
F-Lck	CGGGATCCGCCACCATGGGCTGTTGGTGTTCTTCCAATCCAGAAGACGACAGCGGCCCCGGCA
R-YFP-HRas	TGCTGGCCTCCACCTCTCTTGTACAGCTCGTCCATG
F-YFP-HRas	CTGTACAAGAGAGGTGGAGGCCAGCACAA
R-HRas	GGAATTCCTCAGGAGAGCACACACTTGC
F-Tmem192	CGGGATCCGCCACCATGGCGGCGGGGG
R-link-Tmem192	CCGGGGCCGCTCGTTCTACTTGGCTGACAGC
R-Rheb-YFP	GGAATTCCTCACATCACCGAGCACGAAGACTTCCCTTGTGAAGCTGCCCCGTCCATGCCGCTGCCGGGG
R-Omp25-YFP	TCCACCTCCGCCTCTCTTGTACAGCTCGTCCATG
F-YFP-Omp25	CTGTACAAGAGAGGCGGAGGTGGATCTGG
R-Omp25-YFP	GGAATTCCTCAGAGCTGCTTTCGGTATCTC
F-PEX3	CGGGATCCGCCACCATGCTGAGGTCTGTATGGAATTTTCTG
R-NLRP3-link-PEX3	GTCATTCCTGACCCGGGACCTGATTCCTGTATTTCTCTGATTTTCTTCTGTCCATATTTC
F-PEX3-link-NLRP3	CAGGAATCAGGTCCCGGGTCAGGAATGACCTCCGTGCGGTG
F-PEX26	AGCGGCCCCGGCAGCGGCCGCCAGCTTTGGGACTCT
R-PEX26	CCCTACCCGGTAGAATTCCTCAGTCACGGATGCGGAG
R-YFP-link-PEX26	CAAAGCTGGCGGCCGCTGCCGGGG
F-NLRP3-YFP	CTTTTGTCTTATACTTGGATCCGCCA
F-PEX3long	GTTCAGGTTCTGGAAGTGGCATGACCTCCGTGCGGTG
R-PEX3long	GCCACTTCCAGAACCTGAACCAGATCCTGAGCCCGATCCACTTCCTGACCCGGGACCTGA
F-R258W	CTTCATCCATTGCTGGGAAGTGTCCC
R-R258W	GGGACACTTCCCAGCAATGGATGAAG
F-4xA	ATCTGCGCCGCTGCCGCTGACTACTGCAAGATGTATAGGCGG
R-4xA	GTAGTCAGCGGCAGCGGCGCAGATGCTGATTCTGCTCAGG
R-GOLGB1-YFP	GCTGCGGTTCGCCGCTGCCGGGG
F-YFP-GOLGB1	GCAGCGGCGAACCGCAGCAAAGCTTTTCTG
R-GOLGB1	GGAATTCCCTATAGATGGCCCGTAAAACACAGA
F-PACT	CCAAGCTTGGTACCGAGCTCGGATCCGCCACCATGAAGAGAATTTATGGTAAATACTTGAGGGCA
R-link-PACT	GCCGCTGCCGGGGCCGCTACCTGTGACTCGATGCCAC

F-link-NLRP3	AGCGGCCCCGGCAG
R-YFP-NLRP3	CAGTGTGATGGATATCTGCAGAATTCCTCATCTCTTGTACAGCTCGTC
R-NLRP3(1-686)	TGCTCACCCCCCCTGATCCTCCGGGGCTGTTGTGGAAGAAGC
F-NLRP3(1-686)	CAGCCCCGGAGGATCAGGGGGGGTGAGCAAGGGCGAGGAG
R-NLRP3:F785A_D786R	AGGTCCAGTTCGACCAGTTTCTGAGAGCTGCTCAGCACGCTGCTGATCCTGGCACAGCACTGGTGGCTCAG
F-NLRP3:F810A	CAGAAACTGGTCGAACTGGACCTGAGCGATAACGCTCTGGGCGACGCCGGCATCAGGCTGCTGTG
F-link-TDP43	GAAGCGGCCCCGGCAGCGGCTCTGAATATATTCGGGTAACCGAAG
R-TRE3G-TDP43	AGCGCCTCCCCTACCCGGTAGAATTCCTCAATCGATAAGCTTCATTCCCCAG
R-NLRP3 20ak link	GCCCGATCCTGACCCACTACCAGATCCTGATCCACTACCTGAGCCGCTGCCGGGGCC
F TRE3G:BamHI:ATG	ACACTTTTGTCTTATACTTGGATCCGCCACCATG
GOLGB1 long linker_F	ATCTGGTAGTGGGTCAGGATCGGGCGAACCGCAGCAAAGCTTTTCTG
GOLGB1 long linker_R	AAGCGCCTCCCCTACCCGGTAGAATTCCCTATAGATGGCCCGTAAAACACAGAAT
R long linker	GCCACTTCCAGAACCTGAACCAGATCCTGAGCCCGATCCACTTCCTGACCCGGGACCTGA
F NLRP3 long linker	GTTCAGGTTCTGGAAGTGGCATGACCTCCGTGCGGTG
R NLRP3:stop:EcoRI	CTCCCCTACCCGGTAGAATTCCTCATCTCTTGTACAGCTCGTCCA
EB_ASC_pLenti_F	AGAACACAGGTGTCGTGACGCGGGATCCGCCACCATGGGGCGCGCGCG
EB_mCer_pLenti_R	GAAGTTGGTGGCGCCGCTGCCGCTAGCCTTGTACAGCTCGTCCATGCC
NLRP3 K228A_R	ATGATCTTTCTAGCCAGGATTGTCGCGCCGATGCCGGCAG
NLRP3 K228A_F	GGCGCGACAATCCTGGCTAGAAAGATCAT
NLRP3 227A 228A 229A_R	CATGATCTTTCTAGCCAGGATTGCCGCGGCGATGCCGGCAGCGC
NLRP3 227A 228A 229A_F	ATCGCCGCGGCAATCCTGGCTAGAAAGATCATGCT
