# Supplementary material for: Clustering of NLRP3 induced by membrane or protein scaffolds promotes inflammasome assembly
Source: Nat Commun. 2025 May 27;16:4887. doi: 10.1038/s41467-025-60277-4 (PMC12117088; doi:10.1038/s41467-025-60277-4)
Supplement: Supplementary file 2 — Reporting Summary [file 41467_2025_60277_MOESM2_ESM.pdf]

nature portfolio

Corresponding author(s): Iva Hafner Bratkovic

Last updated by author(s): 5/7/2025

Reporting Summary

Nature Portfolio wishes to improve the reproducibility of the work that we publish. This form provides structure for consistency and transparency in reporting. For further information on Nature Portfolio policies, see our Editorial Policies and the Editorial Policy Checklist.

Statistics

For all statistical analyses, confirm that the following items are present in the figure legend, table legend, main text, or Methods section.

n/a	Confirmed	
☐	☒	The exact sample size (*n*) for each experimental group/condition, given as a discrete number and unit of measurement
☐	☒	A statement on whether measurements were taken from distinct samples or whether the same sample was measured repeatedly
☐	☒	The statistical test(s) used AND whether they are one- or two-sided<br>*Only common tests should be described solely by name; describe more complex techniques in the Methods section.*
☐	☒	A description of all covariates tested
☐	☒	A description of any assumptions or corrections, such as tests of normality and adjustment for multiple comparisons
☐	☒	A full description of the statistical parameters including central tendency (e.g. means) or other basic estimates (e.g. regression coefficient) AND variation (e.g. standard deviation) or associated estimates of uncertainty (e.g. confidence intervals)
☐	☒	For null hypothesis testing, the test statistic (e.g. *F*, *t*, *r*) with confidence intervals, effect sizes, degrees of freedom and *P* value noted<br>*Give P values as exact values whenever suitable.*
☒	☐	For Bayesian analysis, information on the choice of priors and Markov chain Monte Carlo settings
☒	☐	For hierarchical and complex designs, identification of the appropriate level for tests and full reporting of outcomes
☒	☐	Estimates of effect sizes (e.g. Cohen's *d*, Pearson's *r*), indicating how they were calculated

Our web collection on statistics for biologists contains articles on many of the points above.

Software and code

Policy information about availability of computer code

Data collection	ELISA, LDH release and PI uptake were measured with multiplate reader SinergyMx (BioTek) and Gen 5.1.10 software (BioTek). For western blot G-box (Syngene) and Genesnap 7.09 software were used. Flow cytometry was performed using Cytek Aurora (Cytek Biosciences) and SpectroFlo v. 3.1.0 (Cytek Biosciences) software. Confocal imaging was done using LAS AF v. 2.7.22 on TCS SP5 or TCS SP8 (Leica Microsystems). Ultrathin sections for transmission electron microscopy were prepared with Ultramicrotome (Leica EM UC6) and imaged using Philips CM100 electron microscope (Philips, Eindhoven, The Netherlands) equipped with the CCD camera (AMT, Danvers, MA, United States).
Data analysis	Statistical analysis of data was performed using GraphPad Prism 8 software (GraphPad software, Inc.). Graphs were generated with GraphPad Prism 8 software (GraphPad software, Inc.). Flow cytometry data was analyzed with FlowJo v. 10.10.0 (BD Biosciences). LAS X v. 3.7.4 (Leica Microsystems) and Fiji v. 1.54p (NIH) were used for analysis of images obtained using confocal microscope.

For manuscripts utilizing custom algorithms or software that are central to the research but not yet described in published literature, software must be made available to editors and reviewers. We strongly encourage code deposition in a community repository (e.g. GitHub). See the Nature Portfolio guidelines for submitting code & software for further information.

Data

Policy information about availability of data

All manuscripts must include a data availability statement. This statement should provide the following information, where applicable:

- Accession codes, unique identifiers, or web links for publicly available datasets
- A description of any restrictions on data availability
- For clinical datasets or third party data, please ensure that the statement adheres to our policy

All data are included in the Supplementary Information or available from the authors, as are unique reagents used in this Article. The raw numbers for charts and graphs are available in the Source Data file whenever possible. The data generated in this study are provided in the manuscript, Supplementary Information and in the Source Data file. Technical replicates from independent biological repeats have been included in the Source Data for all experiments in the main figures. All unique materials are available from the corresponding author upon request.

Research involving human participants, their data, or biological material

Policy information about studies with human participants or human data. See also policy information about sex, gender (identity/presentation), and sexual orientation and race, ethnicity and racism.

Reporting on sex and gender	N/A
Reporting on race, ethnicity, or other socially relevant groupings	N/A
Population characteristics	N/A
Recruitment	N/A
Ethics oversight	N/A

Note that full information on the approval of the study protocol must also be provided in the manuscript.

Field-specific reporting

Please select the one below that is the best fit for your research. If you are not sure, read the appropriate sections before making your selection.

[x] Life sciences   [ ] Behavioural & social sciences   [ ] Ecological, evolutionary & environmental sciences

For a reference copy of the document with all sections, see nature.com/documents/nr-reporting-summary-flat.pdf

Life sciences study design

All studies must disclose on these points even when the disclosure is negative.

Sample size	No formal sample size calculation was used. Sample sizes were chosen based on the published literature and our experimental practices depending on the technique used. Each experiment included at least three technical replicates when possible.
Data exclusions	No data were excluded from the study.
Replication	Independent biological repeats were performed minimaly two times, mostly at least three times with consistent results. The exact number of replicates is indicated in figure legends.
Randomization	Microscopic images were aquired from randomly selected fields of view.
Blinding	Researchers were not blinded during experiments and data analysis, as the same person typically performed both tasks.

Reporting for specific materials, systems and methods

We require information from authors about some types of materials, experimental systems and methods used in many studies. Here, indicate whether each material, system or method listed is relevant to your study. If you are not sure if a list item applies to your research, read the appropriate section before selecting a response.

Materials & experimental systems

n/a	Involved in the study	
	x	Antibodies
	x	Eukaryotic cell lines
x		Palaeontology and archaeology
	x	Animals and other organisms
x		Clinical data
x		Dual use research of concern
x		Plants

Methods

n/a	Involved in the study	
x		ChIP-seq
	x	Flow cytometry
x		MRI-based neuroimaging

Antibodies

Antibodies used	Anti-ASC (clone 2EI-7, Merck, 04-147, 1:200);<br>Alexa Fluor® 488 goat anti-mouse IgG (H+L) (Invitrogen, A11001, 1:200);<br>Anti-NLRP3 (Cryo-2, Adipogen, AG-20B-0014-C100, 1:1000);<br>Anti-caspase-1 p20 (Casper-1, Adipogen, AG-20B-0042-C100, 1:1000);<br>Anti-ASC (Adipogen, AG-25B-0006-C100, 1:1000);<br>Anti-IL-1β (GeneTex, 1G-GTX74034-100, 1:2000);<br>Anti-β-Actin (8H10D10, Cell Signaling Technology, 1:5000);<br>Anti-α/β-Tubulin (Cell Signaling Technology, 2148, 1:4000).
Validation	All antibodies were validated by manufacturer as indicated on their website.

Eukaryotic cell lines

Policy information about cell lines and Sex and Gender in Research

Cell line source(s)	Human kidney epithelial cells (HEK239T) (ATCC; CRL-11268);<br>Lenti-X™293T (Takara-Bio; 632180);<br>iBMDM from Nlrp3-/- mice were a kind gift from K. A. Fitzgerald, University of Massachusetts Medical School, Worcester, MA, USA
Authentication	No specific authentication method was used.
Mycoplasma contamination	All cell lines were negative for mycoplasma contamination.
Commonly misidentified lines<br>(See ICLAC register)	No commonly misidentified cell lines were used in the study.

Animals and other research organisms

Policy information about studies involving animals; ARRIVE guidelines recommended for reporting animal research, and Sex and Gender in Research

Laboratory animals	C57BL/6JOlaHSd mice were used for isolation of bone marrow.
Wild animals	No wild animals were used in the study.
Reporting on sex	Sex information was not relevant in this study, but 2 female mice were sacrificed for isolation of bone marrow.
Field-collected samples	No field-collected samples were used in the study.
Ethics oversight	Administration of the Republic of Slovenia for Food Safety, Veterinary Sector and Plant Protection of the Ministry of Agriculture, Forestry, and Foods, Republic of Slovenia (Permit no. U34401-11/2023/3).

Note that full information on the approval of the study protocol must also be provided in the manuscript.

Plants

Seed stocks	*Report on the source of all seed stocks or other plant material used. If applicable, state the seed stock centre and catalogue number. If plant specimens were collected from the field, describe the collection location, date and sampling procedures.*
Novel plant genotypes	*Describe the methods by which all novel plant genotypes were produced. This includes those generated by transgenic approaches, gene editing, chemical/radiation-based mutagenesis and hybridization. For transgenic lines, describe the transformation method, the number of independent lines analyzed and the generation upon which experiments were performed. For gene-edited lines, describe the editor used, the endogenous sequence targeted for editing, the targeting guide RNA sequence (if applicable) and how the editor was applied.*
Authentication	*Describe any authentication procedures for each seed stock used or novel genotype generated. Describe any experiments used to assess the effect of a mutation and, where applicable, how potential secondary effects (e.g. second site T-DNA insertions, mosiacism, off-target gene editing) were examined.*

Flow Cytometry

Plots

Confirm that:

- [x] The axis labels state the marker and fluorochrome used (e.g. CD4-FITC).
- [x] The axis scales are clearly visible. Include numbers along axes only for bottom left plot of group (a 'group' is an analysis of identical markers).
- [x] All plots are contour plots with outliers or pseudocolor plots.
- [ ] A numerical value for number of cells or percentage (with statistics) is provided.

Methodology

Sample preparation	Cells were detached and resuspended in PBS with 2.5 mM EDTA.
Instrument	Spectral flow cytometer Aurora with the blue, violet and red lasers (Cytek Bioscience)
Software	SpectroFlo v3.1.0 software (Cytek Bioscience)<br>FlowJo v. 10.10.0 software (BD Biosciences)
Cell population abundance	No sorting was performed in the study.
Gating strategy	After the selection of the population representing macrophages where debris was removed, doublets were excluded and the singlet population was analyzed for YFP fluorescence.

- [x] Tick this box to confirm that a figure exemplifying the gating strategy is provided in the Supplementary Information.
